# Exploring the potential of VGLL3 methylation as a prognostic indicator for intracranial aneurysm with gender-specific considerations

**DOI:** 10.1042/BSR20231374

**Published:** 2024-03-01

**Authors:** Yuchun Liu, Siqi Chen, Enhao Zhang, Yinbin Xu, Xinpeng Deng, Ziliang Hu, Sheng Nie, Yinglu Lin, Yi Huang

**Affiliations:** 1 Department of Neurosurgery, The First Affiliated Hospital of Ningbo University, Ningbo, Zhejiang 315010, China; 2 Laboratory of Neurological Diseases and Brain Function, The First Affiliated Hospital of Ningbo University, Ningbo, Zhejiang 315010, China; 3 Key Laboratory of Precision Medicine for Atherosclerotic Diseases of Zhejiang Province, Ningbo, Zhejiang 315010, China; 4 Department of Neurology, The Second People’s Hospital of Pingyang County, Wenzhou, Zhejiang 325400, China

**Keywords:** CpG island, DNA Methylation, intracranial aneurysm, vestigial-like 3

## Abstract

DNA methylation is widely recognized to play a role in intracranial aneurysm (IA) pathogenesis. We investigated the levels of methylation of vestigial-like 3 (*VGLL3*) in IA and explored its potential as a prognostic indicator. A total of 48 patients with IA and 48 healthy controls were included in the present study. Methylation levels of CpG sites were assessed using bisulfite pyrosequencing, and levels of *VGLL3, TEAD*, and *YAP* in the blood were measured by real-time quantitative polymerase chain reaction testing. *VGLL3* methylation was significantly higher in controls than in IA patients (*P*=0.001), and this phenomenon was more pronounced in females (*P*<0.001). Compared with the control group, the expression levels of *VGLL3* and *TEAD* in the blood of IA patients were significantly increased, while *YAP* was significantly decreased. *VGLL3* methylation was positively correlated with HDL (*P*=0.003) and female Lpa concentration (*r* = 0.426, *P*=0.03), and was also negatively correlated with age (*P*=0.003), APOE (*P*=0.005), and *VGLL3* mRNA expression (*P*<0.001). Methylation and mRNA expression of *VGLL3* may serve as indicators of IA risk in females (AUC = 0.810 and 0.809). *VGLL3* methylation may participate in the pathogenesis of IA by regulating the expression of the *VGLL3/TEAD/YAP* pathway, and its gene methylation and expression levels have IA risk prediction value.

## Introduction

Approximately 3.2% of the general population is affected by intracranial aneurysms (IAs), which are characterized by an outpouching of the intracranial vessel wall [1]. These aneurysms account for 85% of cases of spontaneous subarachnoid hemorrhages (SAHs) [2]. Aneurysmal subarachnoid hemorrhage (aSAH) is a serious ailment linked to significant global rates of death and illness [3]. At the time of aneurysmal rupture, approximately 15% of patients die, and the 30-day mortality rate can reach up to 45% [4]. Furthermore, around half of the individuals who survive aSAH experience irreversible brain damage [5]. IAs are linked to certain risk factors that can be divided into two categories: non-modifiable factors such as hormones, age, sex, and genetic components, and modifiable factors like smoking, excessive alcohol consumption, and hypertension [6,7]. The susceptibility to this illness is significantly influenced by these alterable factors.

Numerous studies have shown that DNA methylation is a prevalent form of epigenetic alteration. Under the influence of DNA methyltransferase (DNMTs), a methyl transfer occurs from S-adenosylmethionine (SAM) to cytosine on CpG dinucleotide. However, DNA methylation is not a static process; it undergoes dynamic changes due to various factors such as age, environment, nutrition, viruses, and other pathogenic factors [8,9]. An increasing number of research papers have emphasized the link between DNA methylation and the emergence of different medical conditions, such as IAs [10,11]. Maimaiti et al. used novel bioinformatics methods, including transcriptomics analysis, machine learning algorithms, genome-wide association studies (GWAS), Mendelian randomization (MR), and summary data-based Mendelian randomization (SMR), to demonstrate the association between DNA methylation-related genes *DNMT3A, MBD2, DNMT1*, and *DNMT3B* and IA susceptibility [12]. Zhou et al. found that DNA methylation levels are associated with the risk of IA in Chinese males [13].

The vestigial-likes (VGLLs) consist of four homologous proteins that serve as mammalian orthologs of Drosophila vestigial gene products. Every member of this protein family has the TONDU domain, which has a vital function in interacting with transcription factors containing transcriptional enhancer activator domain (TEAD) and working as co-factors for TEADs in transcriptional activities. Interestingly, in tumor cells, *VGLL3* specifically contributes to cell growth promotion through its binding to TEAD. The engagement triggers the Hippo pathway, resulting in the subsequent deactivation of Yes-associated protein (YAP)/transcriptional coactivator with PDZ-binding motif (TAZ) [14]. Ye et al. discovered that the loss of function of Ring finger protein 213 (RNF213) activates the Hippo pathway’s YAP/TAZ to promote overexpression of VEGFR2, thereby promoting pathological angiogenesis in Moyamoya disease [15]. Jongshin Kim et al. found that YAP/TAZ play important roles in retinal and cerebral vascular development, as well as vascular barrier maturation, and also actively participate in pathological angiogenesis [16]. Besides, studies have found that down-regulation of YAP/TAZ in vascular smooth muscle cells can trigger the occurrence of aortic aneurysms, and this mechanism may be associated with increased apoptosis of vascular smooth muscle cells [17,18]. The physiological role of *VGLL3* has not yet been fully determined. Nonetheless, there is substantial evidence connecting *VGLL3* to different types of cancers, including but not limited to breast cancer, colon cancer, and lung cancer [19,20]. Ma et al. found that VGLL3 not only inhibits YAP-dependent TEAD transcriptional activity but actively suppresses transcription by recruiting inhibitory complexes to TEAD [21]. Naoto Hori et al. discovered that VGLL3, together with TEAD, activates the Hippo pathway through large tumor suppressor kinase1/2 (LATS1/2) and angiomotin-like 2 (AMOTL2), leading to the inactivation of YAP/TAZ [14]. Considering the existing evidence, we proposed a hypothesis that DNA methylation in the gene promoter region may regulate *VGLL3* expression and participate in the pathological process of IA through the Hippo pathway.

## Materials and methods

### Data from samples and clinical records

The Ethics Committee of Ningbo First Hospital granted ethical approval for this research. Between September 2015 and December 2016, the study enrolled 48 participants diagnosed with IAs (with an average age of 48.08 ± 0.82 years) and 48 controls of the same age and sex (with an average age of 46.63 ± 0.87 years). Patients were diagnosed with IA using either head and neck 320 dynamic state volume computed tomography angiography or cerebrovascular digital subtraction angiography (DSA), in accordance with established classification criteria. The healthy control participants did not have any preexisting cardiovascular diseases, cerebrovascular diseases, liver or kidney diseases, or other serious illnesses, such as malignant tumors. To facilitate analysis, clinical information was gathered, encompassing individuals’ age, gender, as well as plasma concentrations of high-density lipoprotein (HDL), low-density lipoprotein (LDL), triglycerides (TG), total cholesterol (TC), apolipoprotein A1 (ApoA1), apolipoprotein B (ApoB), lipoprotein a (Lp (a)), and apolipoprotein E (ApoE). On the initial day of their hospitalization, blood samples were collected from all individuals. Standard enzymatic methods were employed to measure the levels of HDL, LDL, TG, and TC, which were then evaluated using automatic biochemical analyzers (Olympus AU2700, Japan).

### Pyrosequencing assay

The magnetic bead isolation technique (Lab-Aid 820, Xiamen, China) was used to extract human genomic DNA. A particular section of the *VGLL3* gene’s promoter region, which includes 12 CpG dinucleotides (Chr3 86,937,973-86,991,149), was chosen for DNA methylated pyrosequencing. The sodium bisulfite DNA conversion was performed using the EpiTect Bisulfite kit (Qiagen, Hilden, Germany), which changes unmethylated cytosine residues to uracil while maintaining methylated cytosines. The PyroMark System (Qiagen, Hilden, Germany), a platform for quantitative analysis of DNA methylation, was utilized to conduct DNA methylation analysis. PyroMark Assay Design software (Qiagen, Hilden, Germany) was utilized to design the PCR primers required for the analysis. Regarding the primers employed for the 12 CpG regions of the *VGLL3* gene, the forward primer is 5′-Biotin-GTTTTATAAGGTTGGGGGTGA-3′, the reverse primer is 5′-ACCCCCCCTAATTACCAATCCCT-3′, and the sequencing primer is 5′-ATTACCAATCCCTCC-3′.

### Real-time quantitative polymerase chain reaction (RT-qPCR)

We selected 30 gender- and age-matched IA patients and controls to detect the expression of *VGLL3* mRNA. Total RNA was extracted from blood using TRIzol reagent (Invitrogen, CA, U.S.A.). cDNA was synthesized from RNA using a high-capacity reverse transcription kit from TransGene Biotech in Beijing, China. RT-qPCR was performed on the LightCycler 480 system (Roche, Mannheim, Germany), using SYBR Green SuperMix (TransGene Biotech, Beijing, China). The qPCR primers were as following sequences: *VGLL3* forward primer, 5′-CAGATGCCTATCAGTTGAGT-3′; *VGLL3* reverse primer, 5′-AGGTAGAAGGAACAGTTAGTAG-3′. *TEAD* forward primer, 5′-GCCGTTGCTTATATCGTTAA-3′; *TEAD* reverse primer, 5′-GAGGTGGAATCTGATGGAAT-3′. *YAP* forward primer, 5′-ACAATGACGACCAATAGCTCAGAT-3′; *YAP* reverse primer, 5′-AACGGTTCTGCTGTGAGGG-3′. The sequence of the internal reference gene *ACTB* and the calculation method of mRNA expression have been described in our previous study [13].

### Statistical analyses

Besides the levels of DNA methylation, the clinical data for each group were presented as averages and standard deviation (SD) or numerical values. Pearson or Spearman’s correlation test was used to examine the association between *VGLL3* methylation and clinical data. A *t*-test was used to analyze data that followed a normal distribution, whereas non-parametric tests were employed to assess data that did not follow a normal distribution. In order to assess the precision of *VGLL3* methylation for IA, receiver operating characteristic (ROC) curves were employed to calculate sensitivity and specificity. The statistical analyses were conducted utilizing IBM Corp's SPSS software version 23.0 in Armonk, NY, U.S.A., and GraphPad Prism V9.0 in La Jolla, CA, U.S.A. A threshold of *P*<0.05 was established to determine statistical significance.

## Results

**Figure 1:**
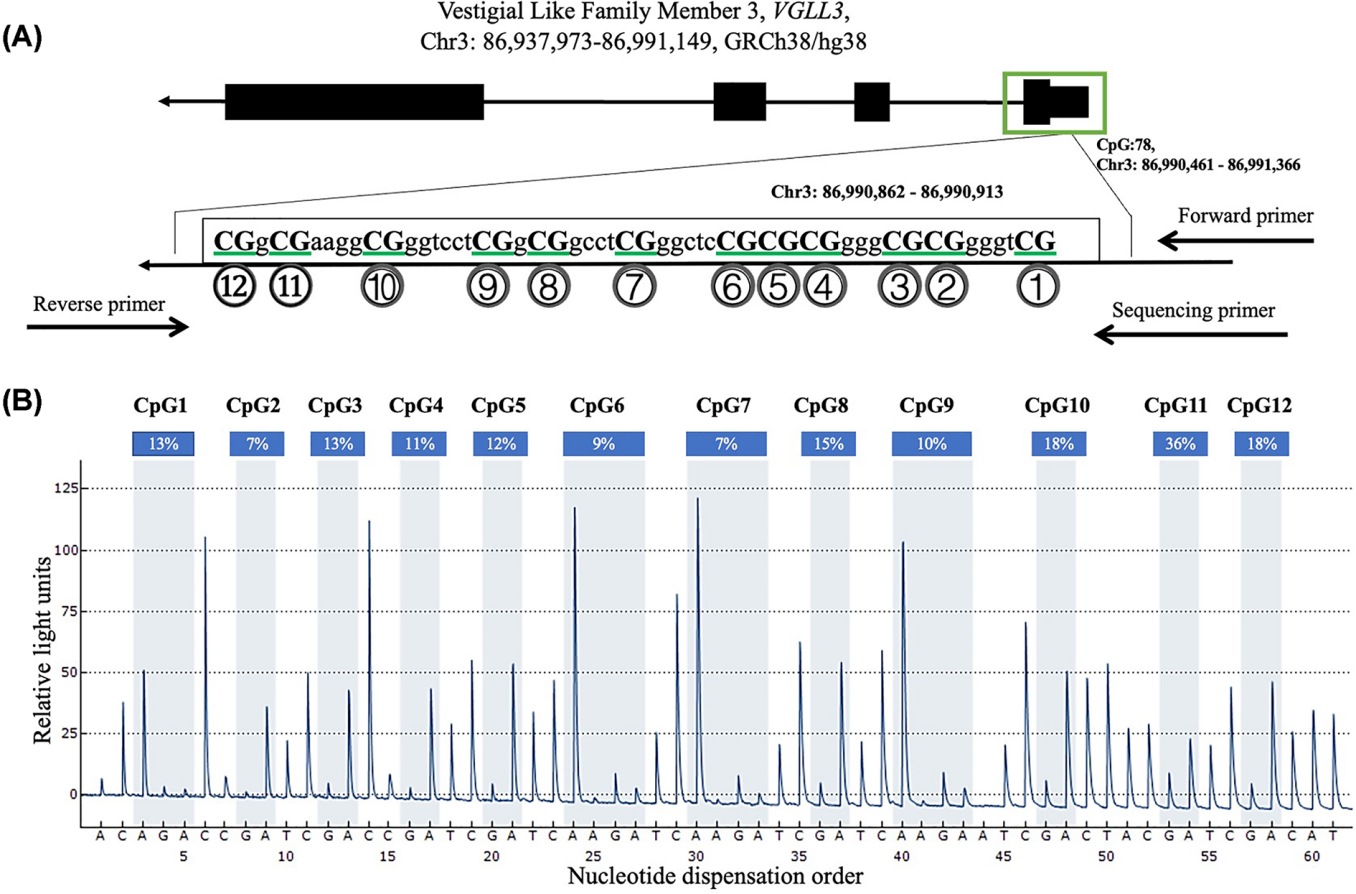
The locations and analysis of the twelve CpGs in *VGLL3* gene (**A**) The locations of the 12 CpGs in *VGLL3* gene. (**B**) Representative sequencing analysis of 12 methylation sites.

**Figure 2:**
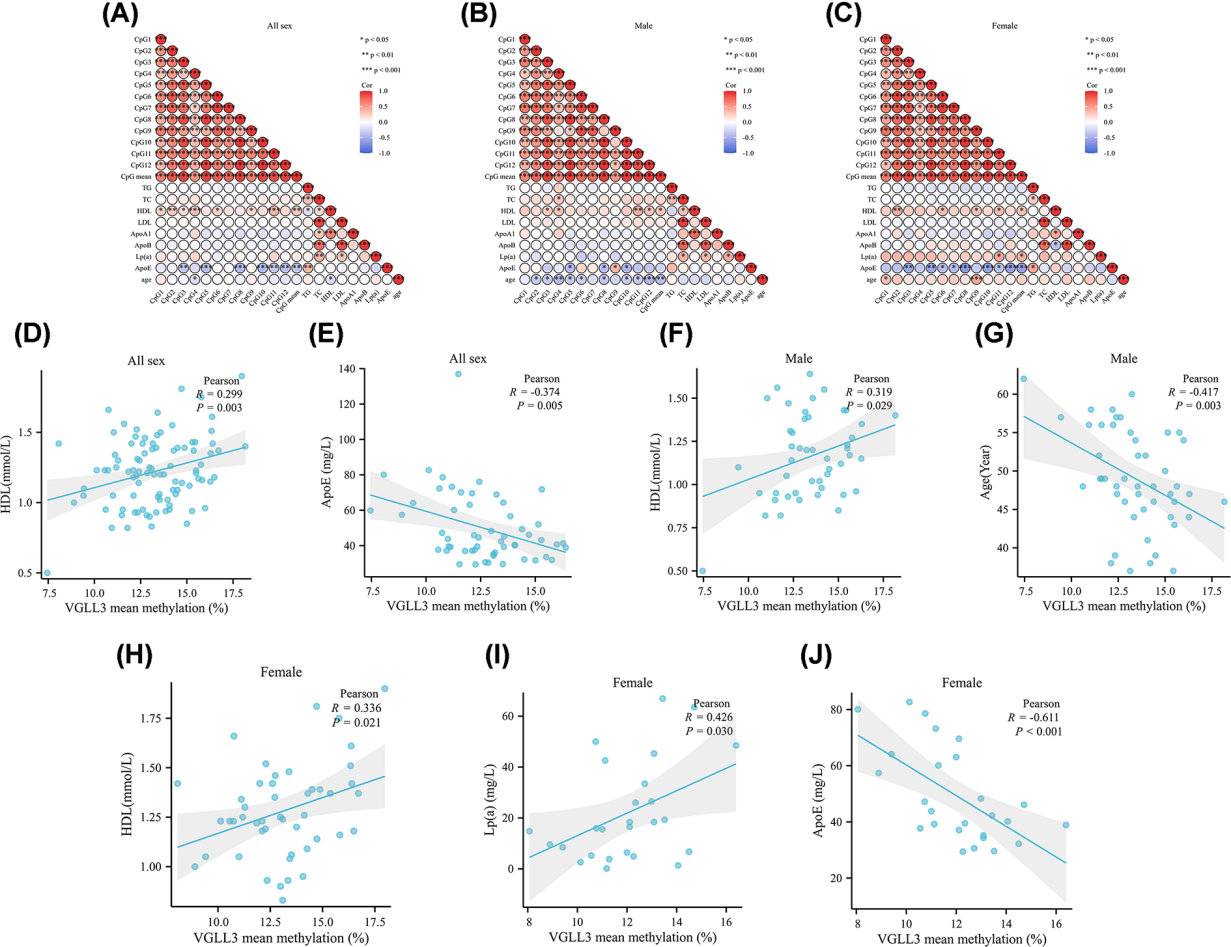
Correlation analysis between *VGLL3* methylation degree and various clinical features (**A**) Correlation analysis in all groups. (**B**) Correlation analysis in all males. (**C**) Correlation analysis in all females. (**D**) Correlation of *VGLL3* methylation with HDL in all sex. (**E**) Correlation of VGLL3 methylation with ApoE in all sex. (**F**) Correlation of VGLL3 methylation with HDL in males. (**G**) Correlation of *VGLL3* methylation with age in males. (**H**) Correlation of *VGLL3* methylation with HDL in females. (**I**) Correlation of *VGLL3* methylation with Lp (a) in females. (**J**) Correlation of *VGLL3* methylation with ApoE in females. Pearson correlation analysis was employed for data analysis.

For this investigation, we chose twelve CpG dinucleotides situated in the promoter region (Chr3 86,990,862-86,990,913) of the *VGLL3* gene to assess the levels of DNA methylation (Figure 1). Significant correlations were observed between the levels of CpG1, CpG2, CpG3, CpG4, CpG6, CpG7, CpG8, CpG9, CpG10, CpG11, CpG12, and the average methylation in males and females, indicating personal exposure (*P*<0.05, Figure 2A–C).

**Figure 3:**
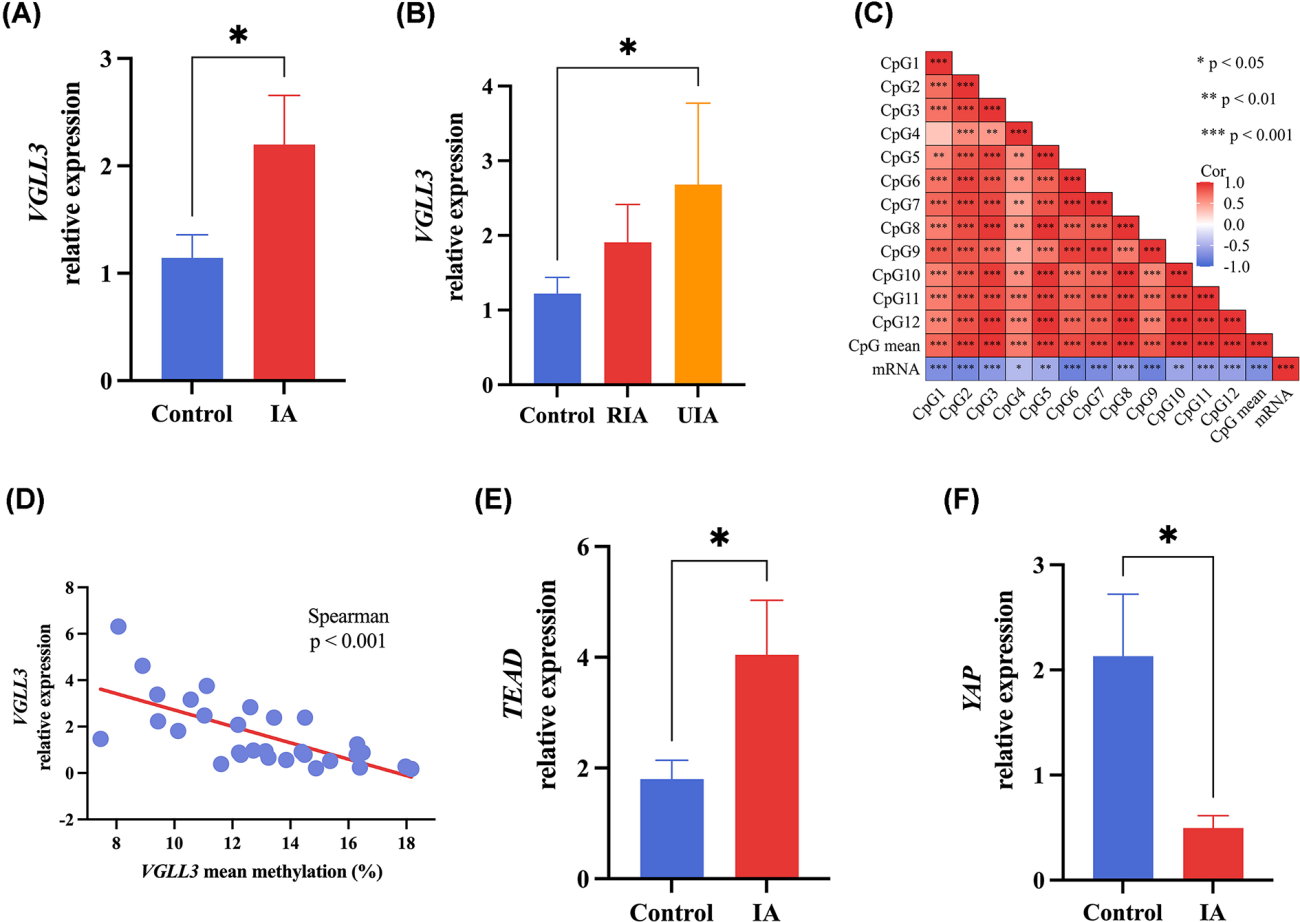
The significant association between *VGLL3* mRNA expression and DNA methylation (**A**) *VGLL3* mRNA expression was much higher in IA than in healthy control. (**B**) *VGLL3* mRNA expression was much higher in UIA patients. (**C**) Correlation between *VGLL3* expression and methylation of each CpG site. (**D**) The *VGLL3* expression was significantly associated with mean DNA methylation in all individuals. (**E**) *TEAD* expression was much higher in IA than in healthy control. (**F**) *YAP* expression was much higher in IA than in healthy control. RIA: ruptured intracranial aneurysm; UIA: unruptured intracranial aneurysm; *P<0.05.

The comparison of clinical data (including age, gender, smoking, drinking, biochemical indicators, etc.) has been reported in previous articles outside, and there was no difference between them [10]. No significant associations were found between *VGLL3* methylation and TG, TC, LDL, ApoA1, ApoB, Lp (a), and age in all sex subjects, based on the clinical data (*P* >0.05). However, in all subjects, HDL showed significant positive correlations with mean methylation (*r* = 0.299, *P*=0.003, Figure 2A,D). Furthermore, there was a notable inverse association between ApoE and mean methylation in individuals (*r* = −0.374, *P*=0.005, Figure 2A,E). In males, HDL exhibited a significant positive correlation with mean methylation (*r* = 0.319, *P*=0.029, Figure 2B,F). In contrast, age exhibited notable inverse associations with mean methylation in males (*r* = −0.417, *P*=0.003, Figure 2B,G). In females, no significant correlations were found between *VGLL3* mean methylation and TG/TC/LDL/ApoA1 or ApoB (Figure 2C). However, HDL showed a significant positive correlation with mean methylation in females (*r* = 0.336, *P*=0.021, Figure 2C,H). Additionally, Lp (a) demonstrated significant positive correlations with mean methylation in females (Figure 2C,I). ApoE also showed significant negative correlations with mean methylation in females (*r* = −0.611, *P*≤0.001, Figure 2C,J). In IA patients, the expression of *VGLL3* mRNA was markedly elevated compared with controls, especially in the unruptured IA group (*P*<0.05, Figure 3A,B). Moreover, the mRNA levels of *VGLL3* exhibited a strong negative correlationwith the extent of DNA methylation enhancement in subjects (*P*<0.05, Figure 3C,D). In addition, we also tested genes related to the Hippo signaling pathway, and the results showed that the expression of *TEAD* mRNA in the blood of IA patients was significantly higher than that of the control group (*P*<0.05, Figure 3E), while the expression of *YAP* gene was lower than that of the control group (*P*<0.05, Figure 3F).

As shown in Table 1, significant differences were observed in CpG2, CpG3, CpG4, CpG5, CpG6, CpG8, CpG10, CpG11, and CpG12 methylation levels between the control and IA groups. Here are the specific results: CpG2: Control vs. IA: 8.65 ± 1.93 vs. 7.39 ± 1.67, *P*=0.001; CpG3: Control vs. unruptured intracranial aneurysm (UIA): 10.72 ± 2.29 vs. 9.50 ± 2.27, *P*=0.036; CpG4: Control vs. IA: 15.64 ± 2.78 vs. 14.09 ± 3.64, *P*=0.021; Control vs. UIA: 15.64 ± 2.78 vs. 13.42 ± 3.17, *P*=0.003; CpG5: Control vs. IA: 11.41 ± 2.41 vs. 9.48 ± 2.60, *P*<0.001; Control vs. ruptured intracranial aneurysm (RIA): 11.41 ± 2.41 vs. 10.02 ± 2.93, *P*=0.034; Control vs. UIA: 11.41 ± 2.41 vs. 8.94 ± 2.15, *P*<0.001; CpG6: Control vs. IA: 8.84 ± 1.84 vs. 7.80 ± 1.55, *P*=0.004; Control vs. RIA: 8.84 ± 1.84 vs. 7.69 ± 1.53, *P*=0.01; Control vs. UIA: 8.84 ± 1.84 vs. 7.92 ± 1.60, *P*=0.04; CpG8: Control vs. IA: 13.41 ± 2.48 vs. 11.46 ± 2.82, *P*=0.001; Control vs. RIA: 13.41 ± 2.48 vs. 11.83 ± 3.14, *P*=0.023; Control vs. UIA: 13.41 ± 2.48 vs. 11.10 ± 2.48, *P*<0.001; CpG10: Control vs. IA: 15.61 ± 2.83 vs. 13.56 ± 3.25, *P*=0.001; Control vs. RIA: 15.61 ± 2.83 vs. 13.81 ± 3.71, *P*=0.025; Control vs. UIA: 15.61 ± 2.83 vs. 13.32 ± 2.78, *P*=0.002; CpG11: Control vs. IA: 36.31 ± 3.47 vs. 34.17 ± 4.08, *P*=0.007; Control vs. RIA: 36.31 ± 3.47 vs. 33.94 ± 3.69, *P*=0.009; CpG12: Control vs. IA: 17.17 ± 2.95 vs. 14.18 ± 3.04, *P*<0.001; Control vs. RIA: 17.17 ± 2.95 vs. 14.31 ± 3.52, *P*=0.001; Control vs. UIA: 17.17 ± 2.95 vs. 14.05 ± 2.53, *P*=0.005. Furthermore, the mean DNA methylation level was significantly different between IA patients and control subjects (12.61 ± 1.94 vs. 13.96 ± 1.99, *P*=0.001).

**Table 1:** DNA methylation difference of twelve CpGs dinucleotides comparison within the *VGLL3* between IAs and Controls

Character	Control (*n*=48)	IA (*n*=48)	RIA (*n*=24)	UIA (*n*=24)	*P* Control vs. IA	*P* Control vs. RIA	*P* Control vs. UIA
CpG1	13.52 ± 2.39	14.03 ± 2.76	14.18 ± 1.82	13.88 ± 3.49	0.332	0.235	0.606
CpG2	8.65 ± 1.93	7.39 ± 1.67	7.52 ± 1.53	7.25 ± 1.83	**0.001**	**0.015**	**0.004**
CpG3	10.72 ± 2.29	9.77 ± 2.39	10.05 ± 2.53	9.50 ± 2.27	0.051	0.259	**0.036**
CpG4	15.64 ± 2.78	14.09 ± 3.64	14.75 ± 4.01	13.42 ± 3.17	**0.021**	0.275	**0.003**
CpG5	11.41 ± 2.41	9.48 ± 2.60	10.02 ± 2.93	8.94 ± 2.15	**<0.001**	**0.034**	**<0.001**
CpG6	8.84 ± 1.84	7.80 ± 1.55	7.69 ± 1.53	7.92 ± 1.60	**0.004**	**0.01**	**0.04**
CpG7	7.21 ± 1.21	6.85 ± 1.06	6.83 ± 0.83	6.87 ± 1.27	0.128	0.173	0.279
CpG8	13.41 ± 2.48	11.46 ± 2.82	11.83 ± 3.14	11.10 ± 2.48	**0.001**	**0.023**	**<0.001**
CpG9	8.97 ± 1.61	8.58 ± 1.73	8.25 ± 1.27	8.91 ± 2.06	0.252	0.059	0.89
CpG10	15.61 ± 2.83	13.56 ± 3.25	13.81 ± 3.71	13.32 ± 2.78	**0.001**	**0.025**	**0.002**
CpG11	36.31 ± 3.47	34.17 ± 4.08	33.94 ± 3.69	34.40 ± 4.51	**0.007**	**0.009**	0.051
CpG12	17.17 ± 2.95	14.18 ± 3.04	14.31 ± 3.52	14.05 ± 2.53	**<0.001**	**0.001**	**0.005**
mean	13.96 ± 1.99	12.61 ± 1.94	12.76 ± 1.90	12.46 ± 2.01	**0.001**	**0.018**	**0.021**

Data were presented as mean ± SD. *P*-value ≤ 0.05 is in bold; RIA, ruptured intracranial aneurysm; UIA, unruptured intracranial aneurysm.

There were notable disparities in the methylation levels of CpG3, CpG4, and CpG10 between male and female subjects across all disciplines (10.84 ± 2.41 vs. 9.66 ± 2.22, *P*=0.014, 13.80 ± 2.80 vs. 15.93 ± 3.47, *P*=0.001, 15.23 ± 3.28 vs. 13.94 ± 3.03, *P*=0.046, Table 2). There was a significant difference in the methylation levels of CpG4 and CpG9 between male and female subjects in the control group (14.33 ± 2.79 vs. 16.95 ± 2.09, *P*=0.001, 8.44 ± 1.19 vs. 9.51 ± 1.82, *P*=0.02, Table 2). Significant differences were also observed in the methylation levels of CpG2, CpG3, CpG5, CpG6, CpG7, CpG8, CpG10, and CpG12 between male and female subjects in IA subjects (8.10 ± 1.63 vs. 6.67 ± 1.42, *P*=0.002; 10.81 ± 2.29 vs. 8.73 ± 2.05, *P*=0.002; 10.43 ± 2.67 vs. 8.53 ± 2.18, *P*=0.010; 8.44 ± 1.60 vs. 7.17 ± 1.23, *P*=0.003; 7.27 ± 1.15 vs. 6.43 ± 0.79, *P*=0.005; 12.52 ± 2.99 vs. 10.38 ± 2.21, *P*=0.007; 14.73 ± 3.36 vs. 12.39 ± 2.74, *P*=0.011; 15.36 ± 3.05 vs. 12.99 ± 2.58, *P*=0.006, Table 2). Accordingly, there was a significant difference between the mean DNA methylation levels of male and female IA patients (13.19 ± 1.84 vs. 13.04 ± 1.90, *P*=0.039, Table 2).

**Table 2:** The gender differences in DNA methylation of 12 CpGs in IAs and Controls, respectively

Character	Male	Female	*t*	*P*
All	48	48		
CpG1	13.81 ± 2.32	13.74 ± 2.85	0.13	0.900
CpG2	8.16 ± 1.70	7.88 ± 2.10	0.69	0.491
CpG3	10.84 ± 2.41	9.66 ± 2.22	2.49	**0.014**
CpG4	13.80 ± 2.80	15.93 ± 3.47	3.3	**0.001**
CpG5	10.86 ± 2.59	10.03 ± 2.72	1.54	0.127
CpG6	8.46 ± 1.61	8.18 ± 1.92	0.767	0.445
CpG7	7.18 ± 1.16	6.88 ± 1.13	1.26	0.212
CpG8	12.98 ± 2.91	11.89 ± 2.64	1.93	0.056
CpG9	8.66 ± 1.35	8.90 ± 1.95	0.7	0.485
CpG10	15.23 ± 3.28	13.94 ± 3.03	2.02	**0.046**
CpG11	34.75 ± 3.69	35.73 ± 4.11	1.22	0.224
CpG12	16.15 ± 3.19	15.20 ± 3.45	1.4	0.165
Mean	13.41 ± 1.98	13.16 ± 2.17	0.57	0.567
Conntrol	24	24		
CpG1	13.09 ± 2.27	13.94 ± 2.49	1.24	0.220
CpG2	8.21 ± 1.80	9.10 ± 1.99	1.62	0.112
CpG3	10.86 ± 2.57	10.58 ± 2.02	0.42	0.680
CpG4	14.33 ± 2.79	16.95 ± 2.09	3.69	**0.001**
CpG5	11.30 ± 2.47	11.52 ± 2.38	0.32	0.752
CpG6	8.48 ± 1.66	9.20 ± 1.97	1.37	0.177
CpG7	7.09 ± 1.18	7.33 ± 1.25	0.71	0.484
CpG8	13.42 ± 2.82	13.39 ± 2.15	0.05	0.964
CpG9	8.44 ± 1.19	9.51 ± 1.82	2.41	**0.020**
CpG10	15.74 ± 3.18	15.48 ± 2.50	0.32	0.754
CpG11	35.58 ± 3.93	37.03 ± 2.84	1.47	0.149
CpG12	16.94 ± 3.19	17.40 ± 2.74	0.54	0.595
mean	13.62 ± 2.12	14.29 ± 1.83	1.16	0.252
IA	24	24		
CpG1	14.53 ± 2.18	13.54 ± 3.21	1.25	0.219
CpG2	8.10 ± 1.63	6.67 ± 1.42	3.24	**0.002**
CpG3	10.81 ± 2.29	8.73 ± 2.05	3.32	**0.002**
CpG4	13.28 ± 2.77	14.90 ± 4.24	1.57	0.123
CpG5	10.43 ± 2.67	8.53 ± 2.18	2.69	**0.010**
CpG6	8.44 ± 1.60	7.17 ± 1.23	3.1	**0.003**
CpG7	7.27 ± 1.15	6.43 ± 0.79	2.93	**0.005**
CpG8	12.52 ± 2.99	10.38 ± 2.21	2.84	**0.007**
CpG9	8.88 ± 1.48	8.29 ± 1.93	1.19	0.241
CpG10	14.73 ± 3.36	12.39 ± 2.74	2.64	**0.011**
CpG11	33.92 ± 3.32	34.42 ± 4.78	0.422	0.675
ÍCpG12	15.36 ±3.05	12.99 ± 2.58	2.89	**0.006**
Mean	13.19 ± 1.84	13.04 ± 1.90	2.13	**0.039**

Data presented are mean ± SD in males and females; *P*-value ≤ 0.05 is in bold.

When stratified by sex, the results generally showed similar trends, with a slightly stronger association in women compared to men. Among male IA patients, the level of CpG1 methylation was notably greater compared with the control group (14.53 ± 2.18 vs. 13.09 ± 2.27, *P*=0.03, Table 3). In contrast, the CpG12 methylation levels were notably reduced in male UIA patients compared with the control group (14.70 ± 2.69 vs. 16.94 ± 3.19, *P*=0.044, Table 3). Nevertheless, there was no notable disparity in the average DNA methylation level among IA patients and the control group (*P*>0.05). Significant variations in the methylation levels of CpG2, CpG3, CpG4, CpG5, CpG6, CpG7, CpG8, CpG9, CpG10, CpG11, and CpG12 were observed in female IA patients when compared with the control group (*P*<0.05, Table 4). Moreover, IA patients showed significantly lower DNA methylation levels (12.04 ± 1.90 vs. 14.29 ± 0.83, *P*<0.001, Table 4).

**Table 3:** The DNA methylation level of 12 CpGs comparison between IAs and Controls in male patients

Character	Control (*n*=24)	IA (*n*=24)	RIA (*n*=12)	UIA (*n*=12)	*P* Control vs. IA	*P* Control vs. RIA	*P* Control vs. UIA
CpG1	13.09 ± 2.27	14.53 ± 2.18	14.43 ± 1.36	14.63 ±2.84	**0.030**	0.07	0.088
CpG2	8.21 ± 1.80	8.10 ± 1.63	8.33 ± 1.10	7.87 ±2.06	0.831	0.831	0.618
CpG3	10.86 ± 2.57	10.81 ± 2.29	11.36 ± 2.29	10.27 ±2.25	0.950	0.573	0.505
CpG4	14.33 ± 2.79	13.27 ± 2.77	14.15 ± 1.64	12.39 ±3.42	0.196	0.845	0.078
CpG5	11.30 ± 2.47	10.43 ± 2.68	11.25 ± 2.84	9.59 ±2.33	0.245	0.959	0.055
CpG6	8.48 ± 1.66	8.44 ± 1.60	8.44 ± 1.48	8.45 ±1.77	0.935	0.937	0.959
CpG7	7.09 ± 1.18	7.27 ± 1.15	7.25 ± 0.79	7.29 ± 1.46	0.587	0.67	0.652
CpG8	13.42 ± 2.82	12.54 ± 2.99	13.25 ± 3.32	11.84 ± 2.57	0.301	0.868	0.112
CpG9	8.44 ± 1.19	8.88 ± 1.48	8.81 ± 1.32	8.94 ± 1.69	0.267	0.398	0.311
CpG10	15.74 ± 3.18	14.73 ± 3.36	15.27 ± 3.72	14.19 ± 3.03	0.291	0.694	0.172
CpG11	35.58 ± 3.93	33.92 ± 3.32	33.79 ± 2.89	34.05 ± 3.83	0.120	0.170	0.275
CpG12	16.94 ± 3.19	15.36 ± 3.05	16.02 ± 3.35	14.70 ± 2.69	0.086	0.428	**0.044**
mean	13.62 ± 2.12	13.19 ± 1.84	13.53 ± 1.62	12.85 ± 2.04	0.453	0.892	0.305

Data presented are mean ± SD between IAs and controls, *P*-value ≤ 0.05 is in bold; RIA, ruptured intracranial aneurysm; UIA, unruptured intracranial aneurysm.

**Table 4:** The DNA methylation level of 12 CpGs comparison between IAs and Controls in female patients

Character	Control (*n*=24)	IA (*n*=24)	RIA (*n*=12)	UIA (*n*=12)	*P* Control vs. IA	*P* Control vs. RIA	*P* Control vs. UIA
CpG1	13.94 ± 2.49	13.54 ± 3.21	13.94 ± 2.22	13.14 ± 4.03	0.626	0.997	0.462
CpG2	9.10 ± 1.99	6.67 ± 1.42	6.71 ± 1.51	6.64 ± 1.38	**<0.001**	**0.001**	**0.001**
CpG3	10.58 ± 2.02	8.73 ± 2.05	8.73 ± 2.09	8.73 ± 2.11	**0.003**	**0.015**	**0.015**
CpG4	16.95 ± 2.09	14.90 ± 4.24	15.35 ± 5.49	14.45 ± 2.66	**0.041**	0.346	**0.004**
CpG5	11.52 ± 2.38	8.53 ± 2.18	8.78 ± 2.55	8.29 ± 1.81	**<0.001**	**0.003**	**<0.001**
CpG6	9.20 ± 1.97	7.17 ± 1.23	6.94 ± 1.22	7.39 ± 1.26	**<0.001**	**0.001**	**0.007**
CpG7	7.33 ± 1.25	6.43 ± 0.79	6.41 ± 0.66	6.45 ± 0.94	**0.005**	**0.024**	**0.039**
CpG8	13.39 ± 2.15	10.38 ± 2.21	10.41 ± 2.28	10.36 ± 2.24	**<0.001**	**0.001**	**<0.001**
CpG9	9.51 ± 1.82	8.29 ± 1.93	7.68 ± 0.98	8.89 ± 2.45	**0.029**	**<0.001**	0.396
CpG10	15.48 ± 2.50	12.39 ± 2.74	12.35 ± 3.22	6.36 ± 1.40	**<0.001**	**0.003**	**0.001**
CpG11	37.03 ± 2.84	34.42 ± 4.78	34.10 ± 4.47	34.74 ± 5.24	**0.027**	**0.022**	0.096
CpG12	17.40 ± 2.74	13.00 ± 2.58	12.59 ± 2.88	13.41 ± 2.30	**<0.001**	**<0.001**	**<0.001**
mean	14.29 ± 0.83	12.04 ± 1.90	12.00 ± 1.91	12.08 ± 1.98	**<0.001**	**0.001**	**0.002**

Data presented are mean ± SD between IAs and controls, *P*-value ≤ 0.05 is in bold; RIA, ruptured intracranial aneurysm; UIA, unruptured intracranial aneurysm.

**Figure 4:**
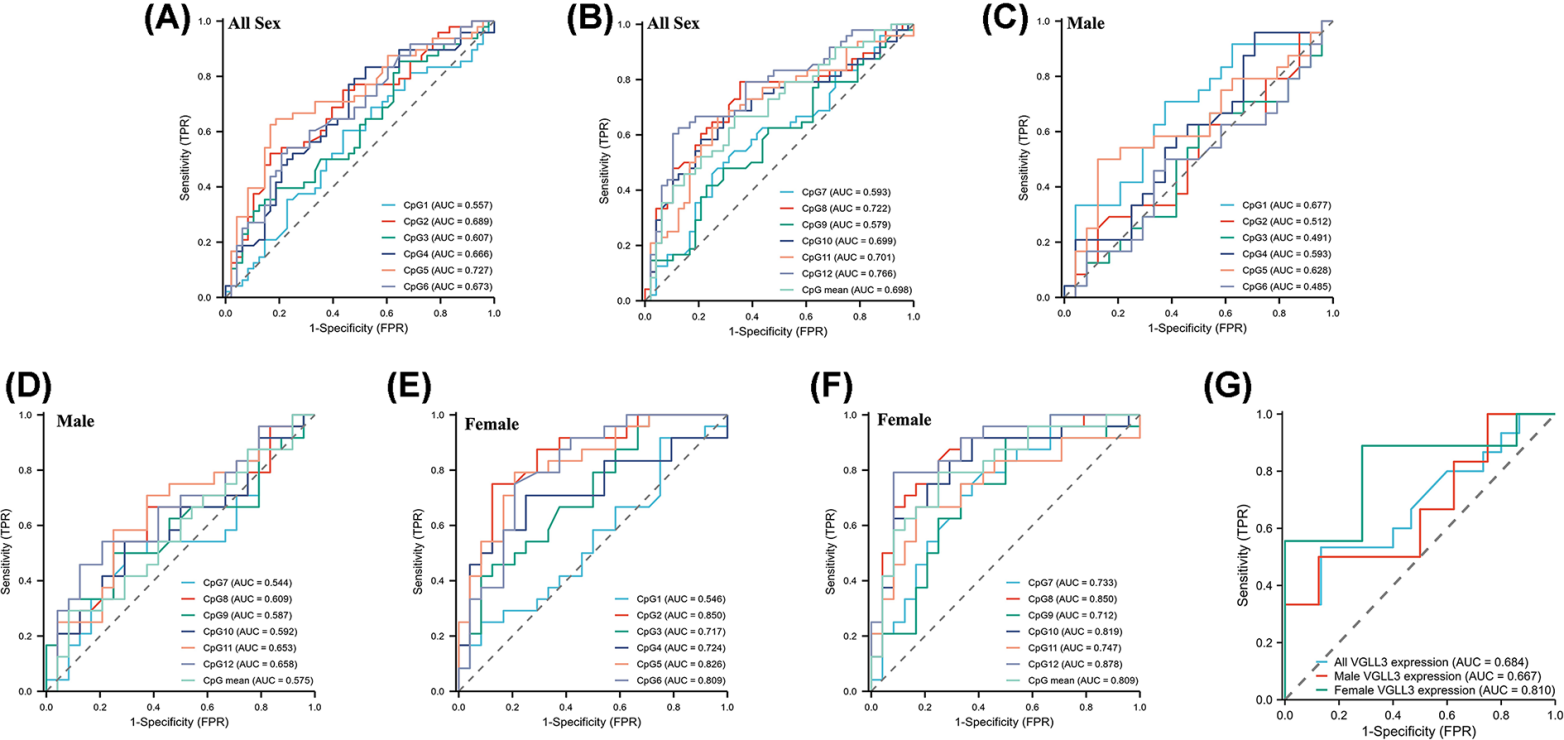
ROC cure of *VGLL3* gene DNA methylation in IA patients (**A,B**) ROC curve for *VGLL3* DNA methylation in all sexes. (**C**,**D**) ROC curve for *VGLL3* DNA methylation in male. (**E,F**) ROC curve for *VGLL3* DNA methylation in females; (**G**) ROC curve for *VGLL3* mRNA expression.

ROC curves were created using GraphPad Prism V9.0 to evaluate the diagnostic effectiveness of *VGLL3* gene DNA methylation in IA patients. As shown in Figure 4A,B, the results are summarized. The area under the curves (AUC) values for CpG5, CpG8, CpG10, CpG11, CpG12, and mean methylation were 0.727, 0.722, 0.699, 0.701, 0.766, and 0.698, in relation to all IA patients of both genders. However, the AUC of *VGLL3* gene DNA methylation in male were <0.70 (Figure 4C,D). Figure 4E,F shows that in IA female patients, the AUC values for CpG2, CpG3, CpG4, CpG5, CpG6, CpG7, CpG8, CpG9, CpG10, CpG11, CpG12, and mean methylation were 0.850, 0.717, 0.724, 0.826, 0.809, 0.733, 0.850, 0.712, 0.819, 0.747, 0.878, and 0.809, respectively. *VGLL3* expression showed a reliable predictor in IA female patients (AUC = 0.810, Figure 4G).

## Discussion

Over the last 10 years, there has been notable advancement in genetic studies of IA, as it has successfully discovered additional genetic susceptibility markers and deepened our comprehension of the disorder [22]. The main objective of our research was to examine the association between methylation of the *VGLL3* gene and the susceptibility to IA in the Chinese Han community. The findings from our study revealed several key insights. *VGLL3* methylation was found to be inversely related to male age at the beginning of the study. Nevertheless, in women, there was no notable association between *VGLL3* methylation and age. Furthermore, it was observed that the IA patients exhibited a decreased methylation level of *VGLL3* in comparison to the control group. This indicates that the decreased methylation of *VGLL3* could potentially be a reliable indicator of the risk of IA, especially in females.

ApoE functions as a carrier protein that interacts with lipids to create lipoprotein particles, thereby playing a role in lipid metabolism and transportation within the central nervous system. In relation to ApoE, Kokubo et al. [23] have reported a positive association between the ε4 allele and subarachnoid hemorrhage (SAH) in eastern Japan. However, other studies have suggested that APOE polymorphism may not be the primary genetic risk factor for SAH or unruptured IAs [24]. The findings of our study demonstrate a negative correlation between serum levels of ApoE and the methylation level of *VGLL3*, as well as a positive correlation between these levels and the expression of *VGLL3* in patients diagnosed with arterial aneurysms. Consequently, we hypothesize that *VGLL3* may play a role in promoting the expression of ApoE. Nevertheless, additional research is warranted to elucidate the mechanistic involvement of ApoE in the pathogenesis of arterial aneurysms. According to reports, a negative correlation was observed between HDL and IA as well as ruptured IA [25]. Additionally, our study revealed a positive correlation between HDL and mean methylation in both males and females. It is possible that VGLL3 may mitigate the incidence of intracranial aneurysms via its interaction with HDL.

**Figure 5:**
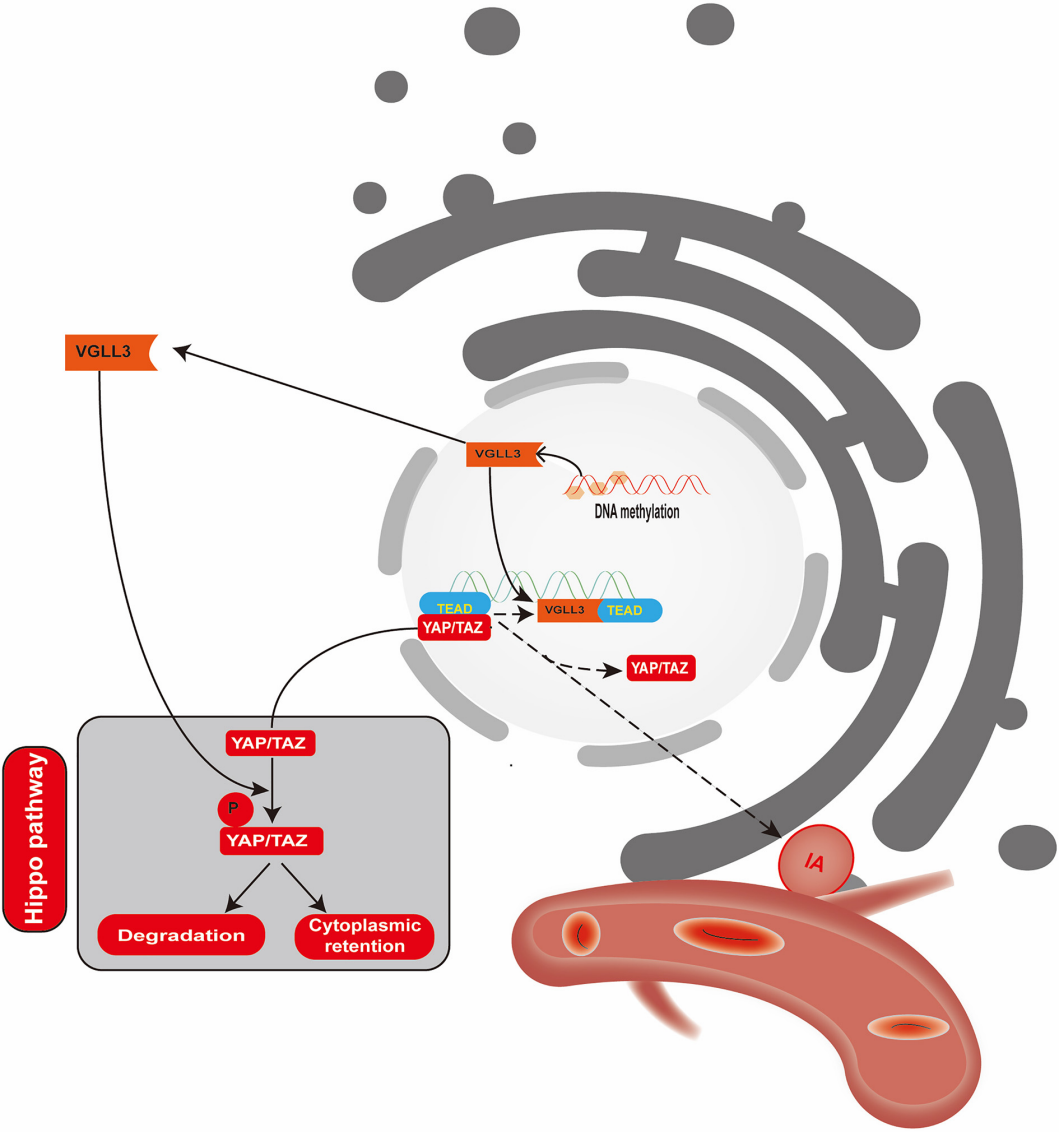
*VGLL3* methylation may participate in the pathogenesis of IA by regulating the expression of the *VGLL3/TEAD/YAP* pathway

*VGLL3* belongs to the vestigial-like protein family and acts as a co-activator in transcription, playing crucial roles in cellular proliferation, differentiation, and development [26]. It interacts with the DNA-binding domain of TEAD, which enhances TEAD’s transcriptional activity. TEAD proteins, which consist of TEAD1-TEAD4, have a broad expression in cells of mammals and play a role in the development of tissues and the formation of tumors [27]. The Hippo pathway plays a vital role in the regulation of organ development and maintaining homeostasis. It functions as a TEAD inhibitor. Disruption of the Hippo signaling pathway may result in excessive tissue growth and the formation of tumors [28]. It has been reported that VGLL3 not only can activate the Hippo signaling pathway to inactivate YAP/TAZ but also can competitively bind with TEAD [18]. The loss of YAP/TAZ function leads to an increase in smooth muscle cell apoptosis and may participate in the occurrence of aortic aneurysm [18]. YAP/TAZ, along with MYC signaling, contributes to the proliferation of endothelial cells, metabolic function, and the control of neovascularization and maturation of the vascular barrier. The irregularity of their function can potentially contribute to abnormal blood vessel formation, which may have a role in the growth and advancement of IAs [16]. According to our study, individuals with IA express significantly more *VGLL3* mRNA than healthy individuals in a control group. Additionally, *VGLL3* expression showed a significant negative correlation with *VGLL3* DNA methylation in all individuals. The expression of *TEAD* in clinical samples is consistent with *VGLL3* expression, while the expression of *YAP* is opposite. These findings suggest that decreased DNA methylation of *VGLL3* may activate the Hippo signaling pathway by increasing the expression of *VGLL3*, leading to a decrease in the nuclear localization of *YAP/TAZ* bound to *TEAD* and ultimately resulting in the formation of IAs (Figure 5).

It has been reported that the expression of *VGLL3* and the stimulation of TGF-β can enhance the secretion of interleukin-1α (IL-1α) and trigger an inflammatory response that depends on NF-KB [29]. Additionally, TEAD1, TEAD3, and TEAD4 have been implicated in the induction of IL-1α by *VGLL3*. The inflammatory response is significantly influenced by NF-κB, which acts as a crucial transcription factor. This protein has been recognized as a regulatory transcription factor for genes linked to IA and could potentially play a role in the rupture of IA [30]. The application of shear stress, a recognized trigger of IA development, has demonstrated the ability to stimulate the activation of proinflammatory genes through a pathway mediated by NF-κB [31]. Moreover, excessive activation of NF-κB results in the production of diverse inflammatory proteins, such as COX-2, PGE-2, and other molecules, which have the potential to promote the advancement of IAs [1]. In addition, NF-κB triggers the production of monocyte chemoattractant protein 1 (MCP-1) and vascular cell adhesion molecule 1 (VCAM-1), which play a crucial role in attracting and binding macrophages [32]. In extracellular matrix remodeling, macrophages also secrete additional proinflammatory substances like tumor necrosis factor-α (TNF-α), interleukin 1-β (IL-1β), metalloproteinases, and proteases [33,34]. As a result, the activation of NF-κB by *VGLL3* may lead to the initiation and progression of IAs, ultimately promoting an inflammatory reaction.

Research has demonstrated that overexpression of *VGLL3* in females can trigger cutaneous and systemic autoimmune diseases resembling systemic lupus erythematosus (SLE) [35]. There is a notable correlation between being female and the creation and growth of IA [36]. The decrease in estrogen levels has been identified as a factor in the development and rupture of IA [37]. Studies conducted recently have revealed disparities between males and females in intricate human ailments and have presented proof that DNA methylation, which can be affected by gender, governs biological mechanisms that could potentially impact human diseases [38]. Studies have recorded disparities in DNA methylation based on gender in genes associated with cardiovascular disorders, lipid processing, lipid concentrations, and the aging process [39]. Our study analyzed the average methylation levels of the *VGLL3* gene in both the control group and the general population. We found no notable distinction between males and females. Nevertheless, we observed a greater vulnerability to methylation in men with IA patients in relation to women, specifically in the *VGLL3* gene. The methylation levels of CpG3 in the *VGLL3* gene were elevated in male IA patients compared with the control group, whereas female IA patients exhibited decreased methylation levels of CpG2/3/4/5/6/7/8/9/10/11/12/mean in comparison with the control group. ROC curve analyses showed that the methylation levels of the *VGLL3* gene could function as a valuable biomarker for diagnosing IA in females, but did not serve as a useful predictor for IA in males.

Transforming growth factor-β (TGF-β) is a type of cytokine that is part of a group of cytokines that have similar structures. TGF-β, which is linked to the development of aneurysms [40], plays a vital part in regulating the growth, specialization, and movement of different cells [41]. Additionally, TGF-β has been observed to have a positive relationship with VEGF levels and promote cellular angiogenesis [42]. Multiple genes in the TGF-β and mitogen-activated protein kinase (MAPK) signaling cascades have been found to be targeted by various microRNAs, such as miR-26b, miR-199a, miR-497, and miR-365. The modulation impacts the inflammatory processes, degradation of extracellular matrix, apoptosis, and the degradation of vascular smooth muscle cells. As a result, it may cause deterioration and bursting of the vessel wall [43]. The extensive deterioration of the extracellular matrix, aided by different collagenases, has been widely acknowledged as a contributing element to the development and bursting of IAs [44]. TGF-β1 is an essential regulatory factor that plays a crucial role in the vascular collagen remodeling process. In different cardiovascular conditions, it promotes the production of collagen and prevents the breakdown of collagen by activating the TGF-β1-Smad2/3 signaling pathway [45]. Studies have additionally found that *VGLL3* has the ability to move into the nucleus via substrate rigidity and experience liquid–liquid phase separation (LLPS). By doing so, it stimulates the synthesis of collagen in fibroblasts found in the heart and liver, consequently contributing to fibrosis in different parts of the body [46].

Apoptosis of cells and the control of smooth muscle cells in blood vessels play a vital role in the proper development and restructuring of the vascular wall. Nevertheless, an overabundance of apoptosis may play a role in the development of vascular-related conditions, such as IAs. The role of intimal vascular smooth muscle cells in the development and breakage of cerebral aneurysms has been recognized [47]. Prior studies have indicated that the p53 gene expression is notably elevated in individuals suffering from IAs. The deregulation of p53 may result in the growth of vascular smooth muscle cells and harm to the vascular wall, consequently encouraging the development and enlargement of aneurysms [48]. Moreover, studies have documented that *VGLL3* can up-regulate p53 and IL-17C to mediate the cellular stress response, ultimately resulting in apoptosis [49].

It is important to acknowledge that our research has a number of constraints. Although our research findings demonstrate strong statistical strength, the sample size utilized in the present study was comparatively limited. Future research should aim to include larger sample sizes, encompassing individuals of various ages and including smokers, to validate and reinforce our conclusions. In addition, our research solely concentrated on 12 distinct GpG nucleotides found in a segment (Chr3 86,937,973-86,991,149) to symbolize the complete promoter area of the *VGLL3* gene. To gain a more thorough comprehension of the methylation patterns within the gene, future research must include a wider array of CpG sites. Third, this study adopts a candidate gene approach, which may result in an incomplete examination of the mechanistic aspects involved. To enhance the validity and reliability of our findings, it is recommended to conduct further investigations utilizing animal models and conducting cellular experiments that can delve deeper into the underlying mechanisms.

To summarize, the decline in *VGLL3* methylation levels is highly linked to the likelihood of developing IAs, especially among females. *VGLL3* methylation may participate in the pathogenesis of IA by regulating the expression of the *VGLL3/TEAD/YAP* pathway, and its gene methylation and expression levels have IA risk prediction value. Nevertheless, additional inquiries are necessary to comprehend the precise mechanisms that underlie the correlation between *VGLL3* and the formation of IAs.

## Data Availability

All supporting data are included within the main article.

## Abbreviations

ApoA1: apolipoprotein A1
ApoB: apolipoprotein B
ApoE: apolipoprotein E
HDL: high-density lipoprotein
IA: intracranial aneurysm
IL-1β: interleukin 1-β
LDL: low-density lipoprotein
Lp (a): lipoprotein a
MCP-1: monocyte chemoattractant protein 1
RIA: ruptured intracranial aneurysm
SAH: subarachnoid hemorrhage
TC: total cholesterol
TG: triglyceride
TNF-α: tumor necrosis factor-α
UIA: unruptured intracranial aneurysm
VCAM-1: vascular cell adhesion molecule 1
